# Metatranscriptomic Analysis Reveals Unexpectedly Diverse Microbial Metabolism in a Biogeochemical Hot Spot in an Alluvial Aquifer

**DOI:** 10.3389/fmicb.2017.00040

**Published:** 2017-01-25

**Authors:** Talia N. M. Jewell, Ulas Karaoz, Markus Bill, Romy Chakraborty, Eoin L. Brodie, Kenneth H. Williams, Harry R. Beller

**Affiliations:** Earth and Environmental Sciences, Lawrence Berkeley National LaboratoryBerkeley, CA, USA

**Keywords:** strain-resolved metatranscriptome, metagenome, aquifer, naturally reduced zone (NRZ), *Hydrogenophaga*, *Bathyarchaeota*, biogeochemistry

## Abstract

Organic matter deposits in alluvial aquifers have been shown to result in the formation of naturally reduced zones (NRZs), which can modulate aquifer redox status and influence the speciation and mobility of metals, affecting groundwater geochemistry. In this study, we sought to better understand how natural organic matter fuels microbial communities within anoxic biogeochemical hot spots (NRZs) in a shallow alluvial aquifer at the Rifle (CO) site. We conducted a 20-day microcosm experiment in which NRZ sediments, which were enriched in buried woody plant material, served as the sole source of electron donors and microorganisms. The microcosms were constructed and incubated under anaerobic conditions in serum bottles with an initial N_2_ headspace and were sampled every 5 days for metagenome and metatranscriptome profiles in combination with biogeochemical measurements. Biogeochemical data indicated that the decomposition of native organic matter occurred in different phases, beginning with mineralization of dissolved organic matter (DOM) to CO_2_ during the first week of incubation, followed by a pulse of acetogenesis that dominated carbon flux after 2 weeks. A pulse of methanogenesis co-occurred with acetogenesis, but only accounted for a small fraction of carbon flux. The depletion of DOM over time was strongly correlated with increases in expression of many genes associated with heterotrophy (e.g., amino acid, fatty acid, and carbohydrate metabolism) belonging to a *Hydrogenophaga* strain that accounted for a relatively large percentage (~8%) of the metatranscriptome. This *Hydrogenophaga* strain also expressed genes indicative of chemolithoautotrophy, including CO_2_ fixation, H_2_ oxidation, S-compound oxidation, and denitrification. The pulse of acetogenesis appears to have been collectively catalyzed by a number of different organisms and metabolisms, most prominently pyruvate:ferredoxin oxidoreductase. Unexpected genes were identified among the most highly expressed (>98th percentile) transcripts, including acetone carboxylase and cell-wall-associated hydrolases with unknown substrates (numerous lesser expressed cell-wall-associated hydrolases targeted peptidoglycan). Many of the most highly expressed hydrolases belonged to a *Ca*. Bathyarchaeota strain and may have been associated with recycling of bacterial biomass. Overall, these results highlight the complex nature of organic matter transformation in NRZs and the microbial metabolic pathways that interact to mediate redox status and elemental cycling.

## Introduction

Shallow aquifers are often characterized as oligotrophic environments in which a heterotrophic microbial lifestyle is supported by surface-derived allochthonous organic matter (Ghiorse and Wilson, 1988), at least in aquifers that are not subject to subsurface anthropogenic contamination. One exception to the generalization of aquifers as oligotrophic is the occurrence of organic-rich, naturally reduced zones (NRZs) in alluvial aquifers, which have been well documented in shallow, alluvial aquifers of the Colorado River Basin, particularly at the U.S. Department of Energy study site in Rifle, CO (e.g., Blazejewski et al., 2009; Campbell et al., 2012; Janot et al., 2015; Wainwright et al., 2016). Such NRZs are of particular interest because they represent biogeochemical hot spots that maintain persistent suboxic/anoxic conditions that can substantially mediate the speciation and mobility of metals and thus influence groundwater geochemistry (Campbell et al., 2012; Janot et al., 2015).

NRZs at the Rifle site are characterized by perennially reduced conditions, fine-grained aquifer sediment, and relative enrichment of organic matter (with prominent woody plant material) and iron sulfide minerals (such as framboidal pyrite and mackinawite) (e.g., Qafoku et al., 2009; Campbell et al., 2012; Janot et al., 2015). NRZs are heterogeneously distributed throughout the Rifle aquifer and are estimated to constitute 10% of the aquifer volume (Qafoku et al., 2009). It has been proposed that NRZs at the Rifle site, which is located on a floodplain adjacent to the Colorado River, were formed as deeply buried riverbank soil horizons (Janot et al., 2015). The buried organic matter (e.g., weathered plant biomass) in these deposits has likely served as a long-lived electron donor fueling sulfate reduction and, directly or indirectly, Fe(III) and U(VI) reduction, thus contributing to the formation of iron sulfide minerals and deposits of poorly soluble U(IV) (e.g., Campbell et al., 2012; Janot et al., 2015).

To some extent, microbial activities in the NRZs have been inferred from extensive geochemical characterization (Campbell et al., 2012; Janot et al., 2015). Although aquifer microbial communities at the Rifle site have been extremely well characterized, particularly through metagenomic (e.g., Castelle et al., 2013; Hug et al., 2013; Brown et al., 2015) as well as metatranscriptomic (Jewell et al., 2016) and proteomic (e.g., Callister et al., 2010) studies, microbial activities in NRZs have not been an explicit focus. Furthermore, the *dynamics* of microbial activity in the NRZs have not been documented with gene expression data or with frequent sampling.

A primary motivation of this study was to investigate, at gene-scale detail, dynamic microbial metabolism in Rifle NRZs. In particular, we were interested in identifying the primary energy sources in these biogeochemical hotspots (e.g., plant material fueling heterotrophic metabolism; iron sulfide minerals fueling chemolithoautotrophic metabolism) and highlighting what components of genomically encoded metabolism were actually being expressed. Thus, in this study, we integrated strain-specific metatranscriptomic and metagenomic data with geochemical data in anaerobic microcosms in which Rifle NRZ sediment served as the sole source of microorganisms and electron donors. We have linked the dominant biogeochemical processes observed during incubation, such as mineralization of dissolved organic carbon (DOC) to CO_2_, followed by a pulse of acetogenesis, with genome-scale information on which metabolic pathways and taxa are catalyzing those activities. Metatranscriptomic data also revealed some highly expressed metabolic activities that would not necessarily be expected for this system and that were not indicated by geochemical data.

## Materials and methods

### Aquifer sediment collection

Sediment samples were collected in March 2013 from a shallow, alluvial aquifer located near Rifle, CO (USA) by waterless sonic drilling (ASTM-D6914-04, 2004) during installation of groundwater monitoring well CMT-03 (Danczak et al., 2016). An extensive site description, including a thorough description of the sonic drilling and sediment sampling process, can be found in Williams et al. (2011). NRZ sediments recovered from a depth profile of 3–4 m below ground surface were placed within N_2_-gassed polyethylene core bags upon recovery from the aquifer and processed within a field-portable anaerobic chamber. Samples from 4-m depth were placed within no-headspace Mason jars and saturated with groundwater pumped from a monitoring well (JB05) proximal to the drilling location (~1.5 m away) to ensure minimal oxygen incursion during storage and shipment. Samples were stored at 4°C until being apportioned into individual microcosms.

### Anaerobic rifle artificial groundwater

Anaerobic Rifle Artificial Groundwater (RAGW) was prepared based on the aqueous geochemical composition of site groundwater [which has been described elsewhere (Williams et al., 2011; Fox et al., 2012)]: 7.7 mM NaHCO_3_, 0.4 mM KCl, 4 mM MgSO^4^.7H_2_O, 4.8 mM CaSO_4_, and 2.6 mM NaCl. As the RAGW did not include a source of N or P, these would have to be provided by the aquifer sediment, as is presumably the case under *in situ* conditions. The basal solution (excluding NaHCO_3_) was made sterile and anaerobic by autoclaving, immediately followed by purging under filtered, anaerobic 90% N_2_—10% CO_2_, using methods described previously (Beller et al., 2012). Anaerobic and sterile sodium bicarbonate (1 M stock solution) was prepared separately in a serum bottle, as described elsewhere (Beller et al., 2012). The bicarbonate stock was added to the artificial groundwater basal solution in an anaerobic chamber (Type B, Coy Laboratory Products, Inc., Grass Lake, Mich.) when both solutions had cooled. The final pH was 7.03. Highly purified water (18Ω resistance) obtained from a Milli-Q Biocel system (Millipore, Bedford, MA) was used to prepare all aqueous solutions described in this article.

### Microcosm construction

Unless otherwise noted, all preparation and sampling of the microcosms were performed within an anaerobic chamber containing a 100% ultrahigh purity N_2_ atmosphere and all equipment (serum bottles, stoppers, forceps, bowl) were sterilized and allowed to degas in the glove box for at least 1 day prior to use. To prepare the inoculum, the overlaying water was removed from the jar containing the aquifer sediment, and the sediment transferred to a sterile stainless steel bowl, where it was homogenized by stirring and breaking up clumps with a sterile stainless steel spatula. Twigs and rocks were manually removed using forceps. In total, 36 microcosms were prepared; details of experimental design are presented in Supplementary Table 1. Twenty four “live” microcosms were prepared with 7 g of the homogenized sediment and 15 mL RAGW in 60-mL serum bottles. The bottles were sealed with butyl rubber stoppers and aluminum crimp-tops, and then protected from light with aluminum foil. The 12 “killed” controls were prepared identically to the live microcosms except that the sediment was autoclaved (121°C for 40 min) prior to the addition of the RAGW. The headspace of each of the 36 microcosms was exchanged by vacuum-gassing three times with ultrahigh purity N_2_. Ten of the live microcosms and four of the killed microcosms were incubated at room temperature, while 10 live microcosms, four killed microcosms, and two sediment-free control microcosms (RAGW only) were attached to a respirometer, also maintained at room temperature. Microcosms were incubated without shaking.

### Microcosm sampling

The microcosm study proceeded for 20 days with sacrificial sampling at days 0, 5, 11, 15, and 20. A sampling scheme detailing the analytical and molecular biological methods that were performed on each microcosm and at which time point is presented in Supplementary Table 1. At time point 0, four live microcosms were immediately sacrificed, and at time points 5, 11, 15, and 20 days, one sacrificial live microcosm was removed from the respirometer and, along with one sacrificial non-respirometer microcosm, was assayed for DOC concentration and δ^13^C_DOC_ (mass spectrometry, or MS); CO_2_, CH_4_, H_2_, O_2_, and N_2_O (gas chromatography, or GC); chloride, acetate, propionate, sulfate, nitrite, and nitrate (ion chromatography, or IC); ${\text{NH}}_{4}^{+}$ and Fe(II) (colorimetric microplate assays), and pH. In addition to the sacrificial sampling, two additional non-respirometer microcosms at each time point were assayed for N_2_O, CH_4_, CO_2_, chloride, acetate, propionate, sulfate, nitrite, nitrate, ${\text{NH}}_{4}^{+}$, DOC, and pH to provide more replicate geochemical analyses. In addition, for metagenomic and metatranscriptomic analysis, one sacrificial live microcosm was removed from the respirometer and, along with one sacrificial non-respirometer microcosm, was preserved for nucleic acid extraction. Killed microcosms were sacrificially collected at days 0 and 20 and were sampled as described above for the live microcosms.

### Respirometry

The Micro Oxymax respirometer (Columbus Instruments, Ohio, USA) was used to measure the evolution of gases (CO_2_ and H_2_, although H_2_ was not detected by the respirometer). The principle of measurement involved gas sampling from the headspace of the sample chamber (i.e., serum bottle). CO_2_ was detected by a single-beam, infrared sensor and H_2_ was detected by an electrochemical sensor. The respirometer attachment had a closed loop that cycled the headspace with periodic measurement of gas evolution every 8 h. It measured both consumption and production rates of gases from the headspace and the net emissions were recorded as accumulated emissions in microliters.

### Analytical methods for gases

In addition to the gas measurements made continuously by respirometry, periodic measurements of CO_2_, CH_4_, N_2_O, H_2_, O_2_, and N_2_ were made by GC. Headspace samples from four microcosms at a given sampling time (Supplementary Table 1) were withdrawn with syringes (4 mL for CO_2_, CH_4_, and N_2_O; 8 mL for H_2_, O_2_, and N_2_ measured in sacrificial samples only) and analyzed on a custom-designed model GC-2014 gas chromatograph (Shimadzu, Pleasanton, CA, USA) equipped with two thermal conductivity detectors (TCD) for CO_2_ (>500 ppm), H_2_, O_2_, and N_2_, an electrical conductivity detector (ECD) for N_2_O, a flame ionization detector (FID) for CH_4_ and CO_2_ (<500 ppm; after conversion to methane in a methanizer), and GCSolution software. Packed, stainless steel columns for gas separations included: 80/100 HayeSep T (1 m × 1/8″ × 2.1 mm ID and 0.5 m × 1/8″ × 2.1 mm ID), 80/100 HayeSep D (2 m × 1/8″ × 2.1 mm ID), and 60/80 Molecular Sieve 5Å (2 m × 1/8″ × 2.1 mm ID). Three-point calibrations were performed using custom gas mixtures (Scott Specialty Gases, Fremont, CA, USA).

### Analytical methods for dissolved constituents

For measurement of dissolved constituents in sacrificial microcosms, the microcosms were transferred to 50-mL tubes, sealed with Parafilm, and centrifuged for 2 min at 14,000 rpm (4°C) outside of the anaerobic chamber. Inside the anaerobic chamber, the supernatant was transferred to a second 50-mL tube and used for assays described below. For dissolved constituents in non-sacrificial microcosms, 1.25 mL of the liquid phase from undisturbed microcosms was removed through the stopper with a syringe and placed in a 1.5 mL Eppendorf tube. The tubes were centrifuged at 14,000 rpm for 2 min (4°C) and the supernatant used in the following assays: the pH was measured using a sympHony pH meter (VWR, Radnor, PA, USA) with an Orion 911600 electrode (Thermo Fisher Scientific, Waltham, MA, USA); ammonium was quantified using a phenol–hypochlorite colorimetric microplate assay modified from Weatherburn (1967); dilutions (1:2 and 1:10) were prepared in reagent water from the remaining supernatant and chloride, acetate, propionate, sulfate, nitrite, and nitrate concentrations were quantified with a model ICS-2000 IC (Dionex, Sunnyvale, CA) as described previously (Beller et al., 2013b).

For DOC and carbon stable isotope composition, 450-μL sample aliquots acidified with 5% (v/v) HCl were loaded and evaporated in 9 × 10 mm Sn capsules (Costech Analytical Technologies, Inc., Valencia, USA). The capsules were then folded and loaded into a zero blank autosampler connected to an ECS 4010 Elemental Analyzer (Costech Analytical Technologies Inc., Valencia, USA) coupled to a Delta V^plus^ isotope-ratio mass spectrometer (Thermo Fisher Scientific, Bremen, Germany). The isotopic composition is reported in the conventional δ^13^C-notation relative to the V-PDB scale. Based upon propionate standards run with sample batches, the analytical precision of carbon concentration and of δ^13^C are estimated at ±2% and ± 0.29%0, respectively.

### Analysis of total Fe(II)

To measure total (dissolved and sediment-associated) Fe(II) in the microcosms, the microcosms were agitated and 0.2 mL of the resulting homogenized slurry was withdrawn through the stopper using a syringe with a 20-gauge needle, immediately dissolved in 1.8 mL of 1N HCl, and vortexed briefly. A colorimetric (ferrozine) microtiter assay for Fe(II) was conducted according to Beller et al. (2013b) with the following modification: the slurry/HCl mixture was incubated for 1 h in the anaerobic glove box and 100 μL of the supernatant after settling was used in the assay.

### Analysis of total organic carbon in sediment

Sediment samples were analyzed for total organic carbon (TOC) by catalytically aided combustion oxidation at 900°C and a NDIR detector using a Shimadzu TOC-V analyzer equipped with a solids module (SSM) on ball-milled sediments after acid fumigation to remove inorganic carbon. Air-dried sediment was ball-milled using tungsten-carbide balls to a fine powder. Acid fumigation with HCl vapors was performed according to Ramnarine et al. (2011).

### Nucleic acid preservation and extraction

Sacrificial microcosms were opened in the anaerobic chamber and the nucleic acids were preserved by mixing 30 mL of RNA preservation reagent (25 mM sodium citrate, 10 mM EDTA, 10 M ammonium sulfate, pH 5.2, Beller et al., 2013a) with the sediment slurry. The preserved sediment slurries were incubated at room temperature for 1 h in the anaerobic chamber, transferred to 50-mL polypropylene centrifuge tubes, removed from the chamber, and centrifuged for 10 min at 8000 × g (4°C). The aqueous layer was discarded and the sediment stored at −80°C until further processing.

Genomic DNA and total RNA were co-extracted and quantified as described by Jewell et al. (2016) with the following modifications: (1) 0.25 g of Chelex-100 (100–200 mesh) (Sigma-Aldrich, CAS Number 11139-85-8) was included with the sediment and extraction buffer to facilitate removal of Fe, and (2) the extracted genomic DNA was further purified with Sera-Mag SpeedBead Carboxyl Magnetic Beads (GE Healthcare, 65152105050250) using a modification of Rohland and Reich (2012), as follows: 1.2X Sera-Mag beads (0.1%, prepared in PEG buffer [18% PEG8000, 1M NaCl, 10 mM Tris-HCl pH 8, 1 mM EDTA pH 8, 0.05% Tween 20 in DEPC-treated water] was added to each sample, mixed by pipetting, and incubated for 10 min at room temperature with gentle tapping every 2 min. The tubes containing the samples were placed on a magnetic holder and incubated 2 min until the Sera-Mag beads separated out of the solution. The supernatant was discarded and the DNA-bound beads washed with 120 μL of 80% ethanol for 1 min. After the ethanol was removed, the DNA was eluted from the beads with TE buffer, placed on the magnetic rack to separate the beads, and the eluent transferred to a fresh tube and stored at −80°C until needed.

### Metagenomic and metatranscriptomic library construction and sequencing

Genomic DNA and cDNA libraries were constructed and sequenced as described by Jewell et al. (2016) and sequenced using an Illumina HiSeq 2500 (San Diego, CA, USA) with 150-bp reads. Paired-end reads were obtained for DNA (ca. 400-bp inserts) and single-end reads for cDNA (ca. 260-bp inserts).

### Analysis of metagenomic and metatranscriptomic short reads

A total of 62.9 Gb from 11 metagenomic samples (5.72 ± 0.46 Gb/sample) were co-assembled, binned, curated, taxonomically and phylogenetically profiled and annotated as described by Jewell et al. (2016). Final co-assembly statistics are given in Supplementary Table 2. A total of 42.99 Gb from 10 metatranscriptomic samples (4.3 ± 2.12 Gb/sample) were mapped separately for each sample to the co-assembly for the quantification of transcripts, as described by Jewell et al. (2016).

### Bioinformatic analysis

Genes of interest for selected metabolic pathways were confirmed with MetaCyc (Caspi et al., 2012) and identified in the metatranscriptome with BLAST 2.2.30+ (Camacho et al., 2009), BLASTP (Altschul et al., 1990), and JGI IMG (Markowitz et al., 2013) as described by Jewell et al. (2016). Additional genes of interest were identified in selected bins using the Department of Energy Systems Biology Knowledgebase (KBase, http://kbase.us) “RAST Annotate Microbial Contigs v1.0.0” workflow. Sequence alignments were performed with MUSCLE (Edgar, 2004), neighbor-joining trees generated using FastTree (Price et al., 2009), and the data were integrated with expression data and visualized with the Interactive Tree Of Life (Letunic and Bork, 2011). Replicate RPKM data were summed for analysis, as were DNA fold coverage data. Heat maps were generated with the R ggplot2 package (Wickham, 2009; R Core Team, 2015).

A data file including ORF IDs and for each ORF: scaffold ID, bin ID, fold coverage from DNA libraries for each sample, RPKM from cDNA libraries for each sample, top 10 UniRef hits, percent protein sequence identities for each UniRef hit, translated sequence of the predicted ORF, functional annotations, and additional information is available at the following doi: http://dx.doi.org/10.21952/WTR/1338826.

## Results

### Temporal profiles of geochemical conditions

**Figure 1:**
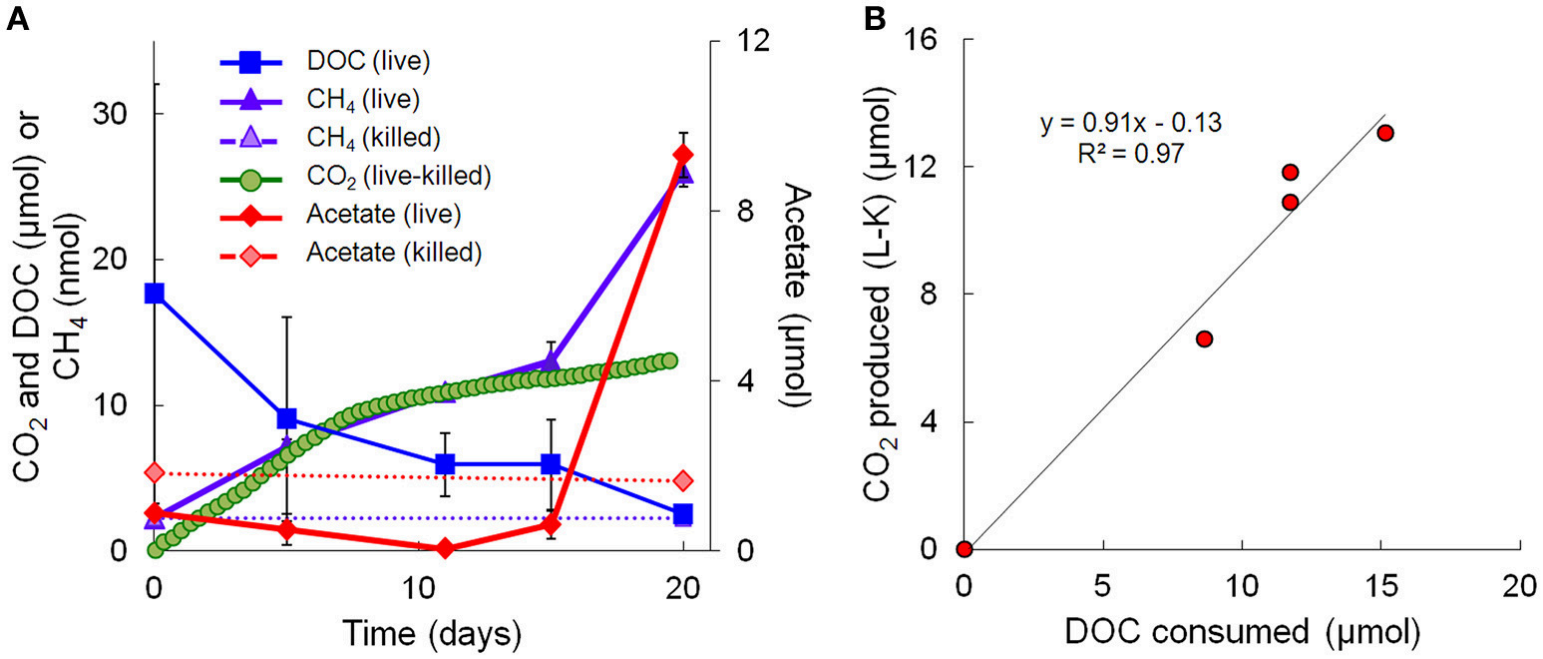
**Geochemical measurements taken during the 20-day microcosm study. (A)** CO_2_, acetate, DOC, and methane. CO_2_ measurements are presented as live minus killed microcosm values to account for abiotic losses of CO_2_ (separate live and killed CO_2_ values are shown in Supplementary Figure 1). **(B)** Linear regression fit of CO_2_ produced vs. DOC consumed. Note that the acetate data on day 20 represents the average of 3 out of 4 replicates—details are given in Table 1.

Geochemical data for the microcosm sediment (1.35 ± 0.03% TOC) and overlaying water were generated over the 20-day time course (Figure 1A; Table 1). The data indicate two phases of carbon metabolism: oxidation of dissolved organic matter (DOM) during the first 15 days and a pulse of acetogenesis and methanogenesis between days 15 and 20. Over the first 15 days, depletion of DOC was strongly correlated to the generation of CO_2_ (*r*^2^ = 0.97, *P* < 0.01; Figure 1B), suggesting biological mineralization of the DOC. During the pulse of acetogenesis and methanogenesis that began at day 15, acetogenesis represented a much greater proportion of transformed carbon than methanogenesis (note the units in Figure 1A). There are several geochemical indicators that the microcosms remained anoxic throughout the experiment, including total Fe(II) (dissolved plus sediment-associated) concentrations, which ranged from 8 to 15 mmol/L from day 0 to 20 (Table 1). Evidence of slight (0.56 mM; 6–7% of initial sulfate) but not significant (*t*-test, *P* > 0.05) sulfate reduction was observed over the 20-day study (Table 1). Abiotic reaction of the resulting hydrogen sulfide with sediment-associated Fe(III) could, at most, have accounted for ~65% of the observed Fe(III) reduction during the study. A full summary of the geochemical data is presented in Table 1.

**Table 1:** **Geochemical data for microcosms over the 20-day study**.

**Variable**	**Live**										**Killed**			
	**Day 0**		**Day 5**		**Day 11**		**Day 15**		**Day 20**		**Day 0**		**Day 20**	
	**Average**	***SD*****^a^**	**Average**	***SD***	**Average**	***SD***	**Average**	***SD***	**Average**	***SD***	**Average**	***SD***	**Average**	***SD***
pH	7.9	0.0	8.1	0.2	8.1	0.2	8.1	0.4	NA^b^	NA	7.9	0.0	NA	
Fe(II) (mM)	8.0	2.4	9.0	0.03	9.8	0.03	9.1	0.01	15.0	2.2	4.5	0.4	NA	
Ammonium (μM)	178	1.6	153	12	143	23	154	17	137	3.3	202	6.1	212	18
Chloride (mM)	3.34	0.13	3.27	0.09	3.29	0.12	3.50	0.21	3.29	0.23	3.36	0.12	3.38	0.23
Acetate (μM)	60	4.3	44	13	6.7	2.8	42	22	620^c^	35^c^	120	26	110	35
Propionate (μM)	<DL^d^		< DL		<DL		< DL		<DL		<DL		<DL	
Sulfate (mM)	8.67	0.11	8.36	0.46	8.26	0.59	8.66	0.90	8.10	0.86	9.00	0.31	8.75	0.39
Nitrate (μM)	114	5.6	118	12	111	14	98	41	120	25	115	2.5	109	17
Nitrite (μM)	<DL		<DL		<DL		<DL		<DL		<DL		<DL	
DOC (μmol)	17.7	14.4	9.1	7.0	5.9	2.2	5.9	3.1	2.5	0.6	69.3	10.5	57.5	7.9
DOC δ^13^C (%0)	−28.54		< DL		<DL		<DL		<DL		−23.82	0.44	−23.56	0.19
Nitrous oxide (ppm)	< DL		< DL		< DL		<DL		<DL		<DL		<DL	
Methane (ppm)	1.3	0.0	4.3	0.29	6.4	0.19	7.7	0.76	15.4	0.56	1.3	0.0	1.3	0.0
Hydrogen (ppm)	<DL		<DL		<DL		<DL		<DL		<DL		<DL	

a *Standard deviation*.

b *Not analyzed*.

c *Note that the acetate data on day 20 (live) represents the average of 3 replicates that were attached to the respirometer; a fourth sample, not attached to the respirometer, did not show an increase in acetate. If all replicates were included, acetate would be 470 ± 310 μM*.

d *Below detection limit: propionate, 10 μM; nitrite, 10 μM; DOC δ^13^C, 0.9 μmol C; nitrous oxide, 0.2 ppm; hydrogen, 40 ppm*.

### Temporal profiles of microcosm metagenomes and metatranscriptomes

**Figure 2:**
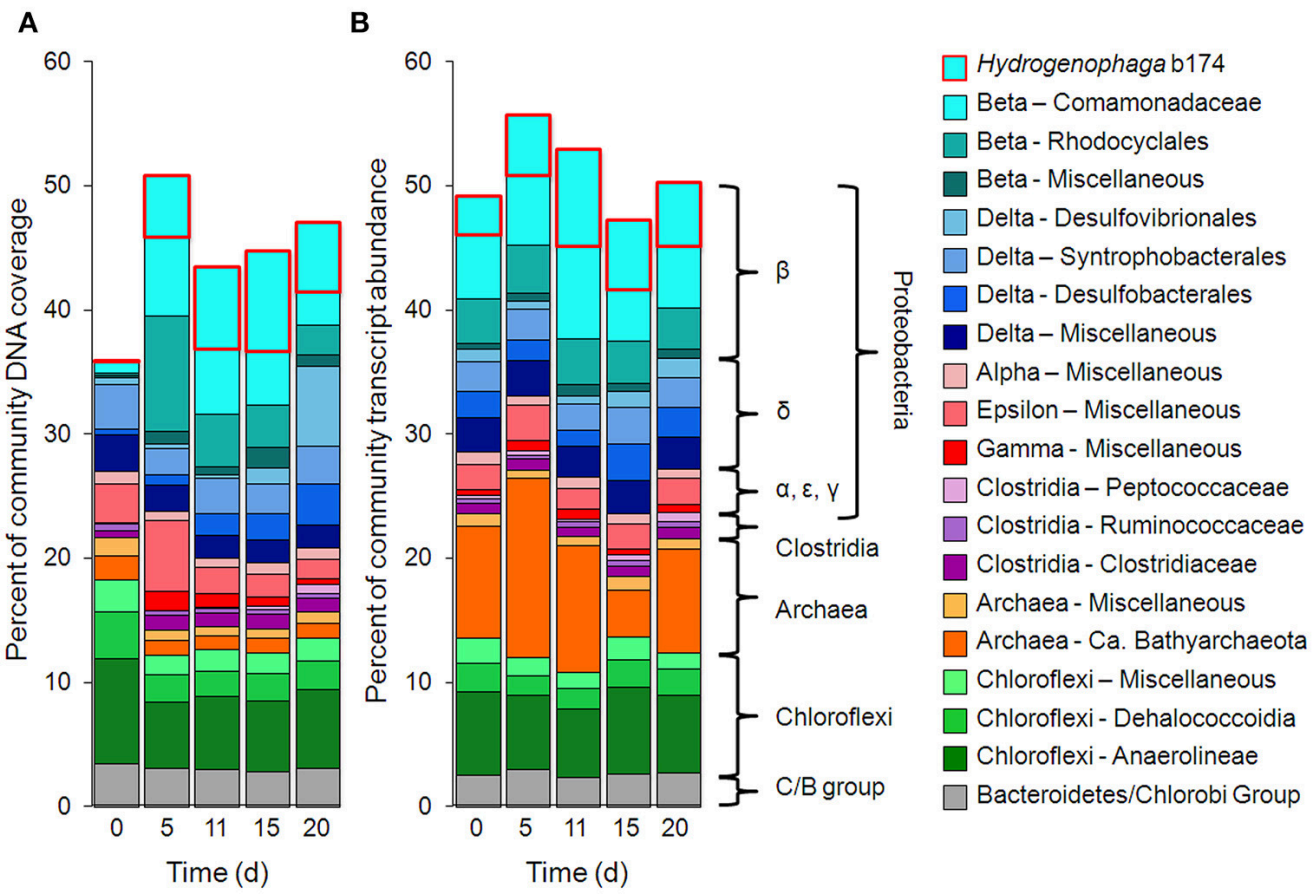
**Primary taxa composition. (A)** Percent of total community mapped DNA (as depth of coverage) and **(B)** percent of total community mapped mRNA (as RPKM). *Hydrogenophaga* b174, belonging to the Comamonadaceae family, is boxed in red.

Metagenomic and metatranscriptomic data for each of the five “live” time points (days 0, 5, 11, 15, and 20) and one “killed” time point (day 20) were analyzed for community composition (percent community DNA abundance) and activity (percent community transcript abundance). The data are available in the Supplementary Information. Bins representing ~50–55% of the DNA and mRNA belonged to the following five taxonomic groups (Figure 2): Clostridia (Clostridiaceae, Ruminococcaceae, and Peptococcaceae), Archaea (*Ca*. Bathyarchaeota and unresolved Archaeal taxa), Chloroflexi (Dehalococcoidia, Anaerolineae, and unresolved Chloroflexi taxa), and the Bacteroidetes/Chlorobi group. The Chloroflexi families accounted for ca. 15% of the metagenome at day 0, comparable to the fraction of Chloroflexi identified in another metagenomic study at the Rifle site under similar conditions (Hug et al., 2013). The fifth group, Proteobacteria, was composed of the subphyla alpha (unresolved taxa), beta (Rhodocyclales, Comamonadaceae, and unresolved taxa), unresolved gamma taxa, delta (Desulfobacterales, Desulfovibrionales, Syntrophobacterales, and unresolved delta taxa), and unresolved epsilon taxa (Figure 2, Supplementary Table 3). Individual bins associated with sulfate-reducing and Fe(III)-reducing bacteria, such as Desulfobacterales, Desulfovibrionales, and *Geobacter* among the delta Proteobacteria, constituted a relatively small portion of the metagenomes and metatranscriptomes in all samples. For example, the most abundant sulfate-reducing bin (b152, representing Desulfovibrionales), composed at most 6.2% of metagenomes and 1.3% of metatranscriptomes across all samples; the most active sulfate-reducing bin (b98, representing Desulfobulbaceae) composed at most 2.2% of metagenomes and 1.7% of metatranscriptomes. However, collectively, bins representing sulfate-reducing bacteria (b1, b7, b16, b37, b71, b84, b93, b98, b103, b140, b152) constituted up to 13% of metagenomes and 6.6% of metatranscriptomes. The most highly expressed dissimilatory sulfite reductase subunits (*dsrA* and *dsrB*) belonged to b98 (k101_1599993_66261_1 and k101_233918_9973_2, respectively). *Geobacter* spp., Fe(III)-reducing bacteria whose presence and activity is well documented at the Rifle site under some conditions (e.g., Anderson et al., 2003), composed less than 0.7% of metagenomes and 0.5% of metatranscriptomes in this study.

**Figure 3:**
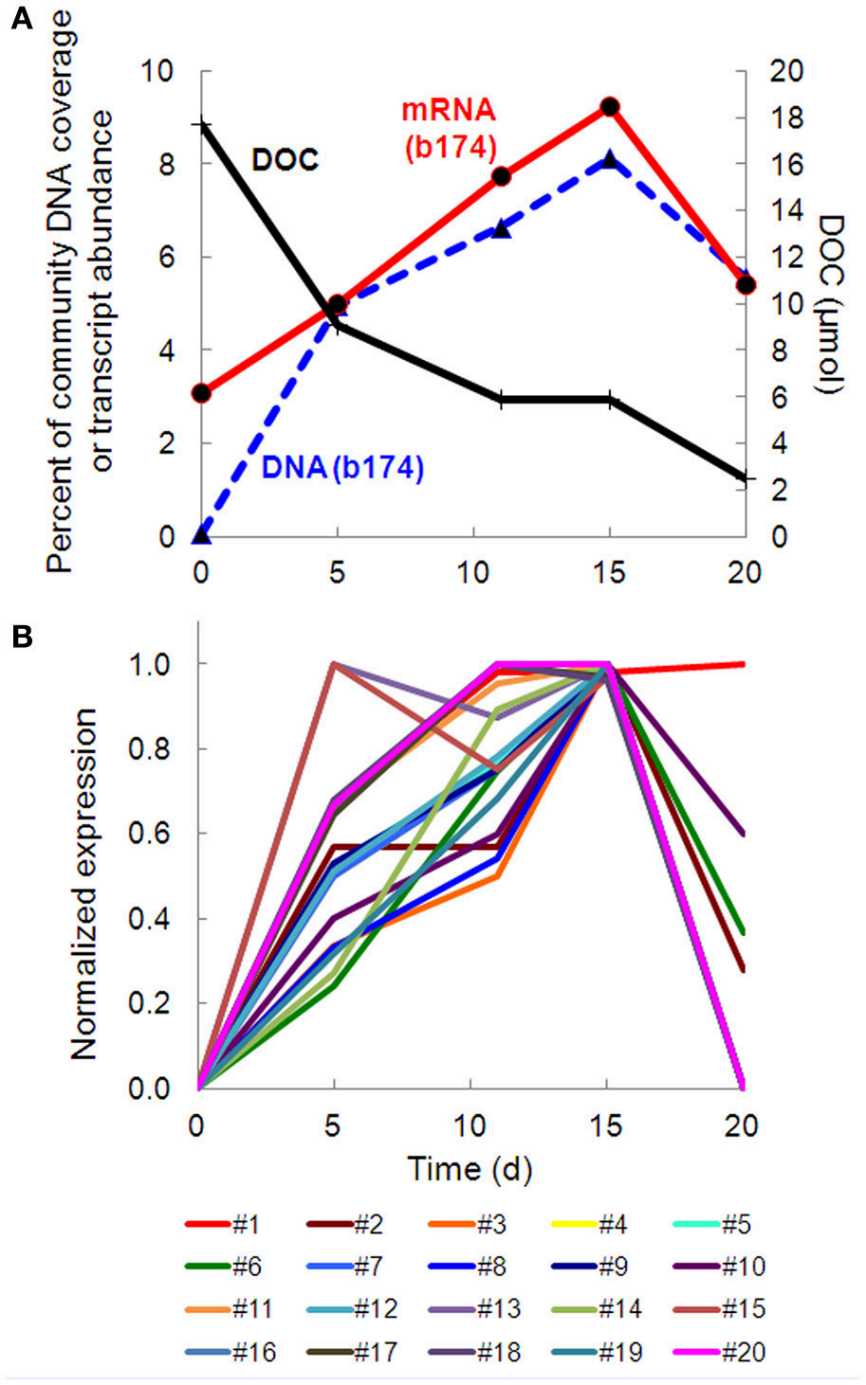
**Temporal trends of *Hydrogenophaga* b174 gene expression and DOC consumption. (A)** Trends of DOC concentration and *Hydrogenophaga* b174 relative population and activity (as % of community DNA and mRNA, respectively). The DOC plot is identical to that in Figure 1. From linear regression analysis, the *r*^2^-value of DOC vs. percent DNA from days 0 to 15 is 0.98 (*P* < 0.05) and of DOC vs. percent mRNA is 0.90 (*P* > 0.05). **(B)** Selected *Hydrogenophaga* b174 ORFs involved in heterotrophic metabolism that have a strong inverse correlation with DOC concentration (*r*^2^ > 0.8 at from days 0 to 15). The ORF annotations are as follows: **fatty acid beta-oxidation**: enoyl-CoA hydratase (#1); **amino acid degradation**: putative amino acid ABC transporter permease (#2), branched-chain amino acid ABC transporter permease (#3), amino acid dehydrogenase (#4), peptidases (#5 and #6); **miscellaneous hydrolases**: alpha/beta hydrolase fold protein (#7), urea carboxylase (#8), allophanate hydrolases (#9 and #10), hydrolases (#11 and #12), hydroxyacylglutathione hydrolase (#13), lytic transglycosylase (#14); **carbohydrate catabolism**: phosphofructokinase (#15), phosphoglycerate kinase (#16), mannose-1-phosphate guanylyltransferase/mannose-6-phosphate isomerase (#17), D-hexose-6-phosphate mutarotase (#18), carbohydrate-binding protein (#19), and malate synthase G (#20). ORF identification, annotation, RPKM, and *r*^2^-values are listed in Supplementary Table 4.

Taxonomic bins (b20, b60.1, b60.2, b117, b137, b174) belonging to the beta-proteobacterial Comamonadaceae family accounted for 15.2% of the metatranscriptome on day 11, with *Hydrogenophaga* bin 174 alone accounting for 7.8%. Abundance (DNA) and metabolic activity (mRNA) of this bin correlated well with DOC consumption (*r*^2^ = 0.97 and 0.75, respectively), suggesting that heterotrophic activity catalyzed by *Hydrogenophaga* was a substantial contributor to DOC mineralization (Figure 3A). The expression of genes relevant to heterotrophic metabolism—including those involved in the catabolism of amino acids, sugars, and fatty acids—also correlated well with DOC consumption (in Figure 3B, correlations of each transcript vs. DOC consumption had *r*^2^-values greater than or equal to 0.85; Supplementary Table 4).

### Metabolic activity of prominent strain, *Hydrogenophaga* b174, as assessed by transcriptional data

**Figure 4:**
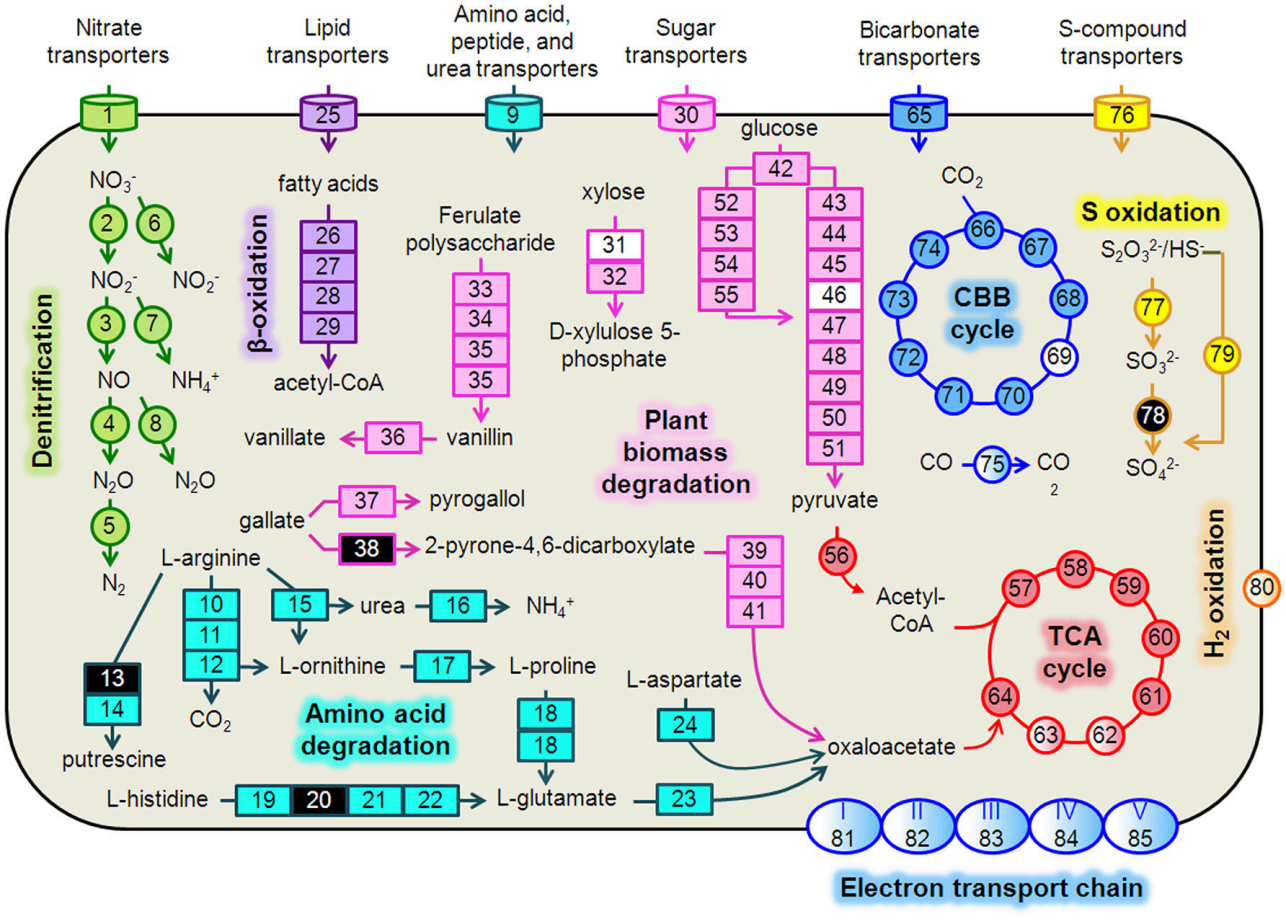
***Hydrogenophaga*** **b174 cell diagram representing selected catabolic and energy conservation pathways active during the study**. This reconstruction is a composite of pathways that were active at any measured point during the 20-day study. Genes that were not expressed, or for which the gene was not identified in the metagenome and therefore no expression level could be assigned, are represented in the diagram as white and black, respectively. Protein complexes (e.g., ETC and hydrogenases) are represented in the diagram with a color gradient if not all subunit genes were expressed. **Denitrification:** 1, nitrate/nitrite transporters (*narK*_*1*_*K*_*2*_); 2, nitrate reductase (*narGH*); 3, nitrite reductase (*nirS*); 4, nitric oxide reductase (*norB*); 5, nitrous oxide reductase (*nosZ*); 6, assimilatory nitrate reductase (*nasAB*); 7, assimilatory nitrite reductase (*nirB*); 8, anaerobic nitric oxide reductase flavoredoxin (*norV*); **Amino acid degradation:** 9, amino acid transporters; 10, arginine deiminase; 11, ornithine carbamoyltransferase; 12, carbamate kinase; 13, arginine decarboxylase; 14, agmatinase; 15, arginase; 16, urease (*ureABC*); 17, ornithine cyclodeaminase; 18, bifunctional proline dehydrogenase/L-glutamate gamma-semialdehyde dehydrogenase; 19, histidine ammonia-lyase; 20, urocanase; 21 imidazolonepropionase; 22, formimidoylglutamase; 23, glutamate dehydrogenase; 24, aspartate aminotransferase; β**-oxidation:** 25, Fatty acid transporters; 26, acyl-CoA dehydrogenase; 27, enoyl-CoA hydratase; 28, 3-hydroxyacyl-CoA dehydrogenase; 29; acetyl-CoA acetyltransferase (thiolase); **Plant biomass degradation:** 30, sugar transporters; 31, xylose isomerase; 32, xylulokinase; 33, tannase and feruloyl esterase; 34, feruloyl-CoA synthase; 35, vanillin synthase /trans-feruloyl-CoA hydratase; 36, vanillin dehydrogenase; 37, pyrogallol hydroxytransferase large subunit; 38, gallate dioxygenase; 39, 2-pyrone-4,6-dicarboxylate hydrolase; 40, 4-oxalomesaconate hydratase; 41, 4-carboxy-4-hydroxy-2-oxoadipate aldolase/oxaloacetate decarboxylase; **Glycolysis**: 42, glucokinase; 43, glucose-6-phosphate isomerase; 44, 6-phosphofructokinase; 45, fructose-1,6-bisphosphate aldolase; 46, triose-phosphate isomerase; 47, type I glyceraldehyde-3-phosphate dehydrogenase; 48, phosphoglycerate kinase; 49, phosphoglycerate mutase; 50, enolase; 51, pyruvate kinase; **Entner-Doudoroff Pathway:** 52, glucose-6-phosphate dehydrogenase; 53, 6-phosphogluconolactonase; 54, phosphogluconate dehydratase; 55, 2-keto-3-deoxy-6-phosphogluconate aldolase; **TCA Cycle:** 56, pyruvate dehydrogenase (acetyl-transferring) (*aceE*); 57, citrate (Si)-synthase (*gltA*); 58, aconitate hydratase (*acnAB*); 59, isocitrate dehydrogenase (NADP^+^) (*icdA);* 60, 2-oxoglutarate dehydrogenase subunit E1 (*sucA*); 61, succinate-CoA ligase subunit, beta (*sucCD*); 62, succinate dehydrogenase flavoprotein subunit (*sdhABCD*); 63, fumarate hydratase (*fumCA*); 64, malate dehydrogenase (*mdh*); **CBB Cycle**: 65, bicarbonate transporters; 66, ribulose-1,5-bisphosphate carboxylase/oxygenase (RubisCO) (*rbcLSQO*); 67, phosphoglycerate kinase; 68, glyceraldehyde-3-phosphate dehydrogenase; 69, triose-phosphate isomerase; 70, fructose-bisphosphate aldolase; 71, fructose-1,6-bisphosphatase class 1; 72, transketolase; 73, ribulose-phosphate 3-epimerase; 74, phosphoribulokinase; 75, “aerobic” carbon monoxide dehydrogenase (*coxLSM*); **S oxidation:** 76, S-compound transporters; 77, rhodanese; 78, sulfite dehydrogenase; 79, S oxidation genes (*soxCD*, cytochrome *c*_552_, cytochrome *c*_551_, *soxYZAXBR*); **H**_2_ **oxidation:** 80, [NiFe] hydrogenase (*hupLU*) and accessory/maturation proteins; **electron transport chain (ETC)/oxidative phosphorylation:** 81, ETC complex I—NADH:ubiquinone oxidoreductase (*nuoABCDEFGHIJKLMN*); 82, ETC complex II—succinate dehydrogenase (*sdhCD, sdhAB*); 83, ETC complex III—cytochrome *bc*_1_-type ubiquinol oxidoreductase (*petAB*); 84, ETC complex IV—*cbb*_3_-type cytochrome *c* oxidase (*ccoPQON*); 85, ETC complex V—ATP synthase (*atpCDIBEFHAG*).

We reconstructed expressed metabolic pathways in *Hydrogenophaga* b174 and found evidence of a surprisingly diverse lifestyle (Figure 4, Supplementary Table 5). *Hydrogenophaga* b174 appears to be a facultatively denitrifying, mixotrophic strain that can assimilate CO_2_ autotrophically through the Calvin-Benson-Bassham pathway (which includes RubisCO) and has numerous means of catabolizing plant-derived compounds for heterotrophic growth. Electron donors for chemolithoautotrophy included H_2_, CO, and reduced S compounds, as discussed in more detail below.

#### Denitrification and electron transport phosphorylation

**Figure 5:**
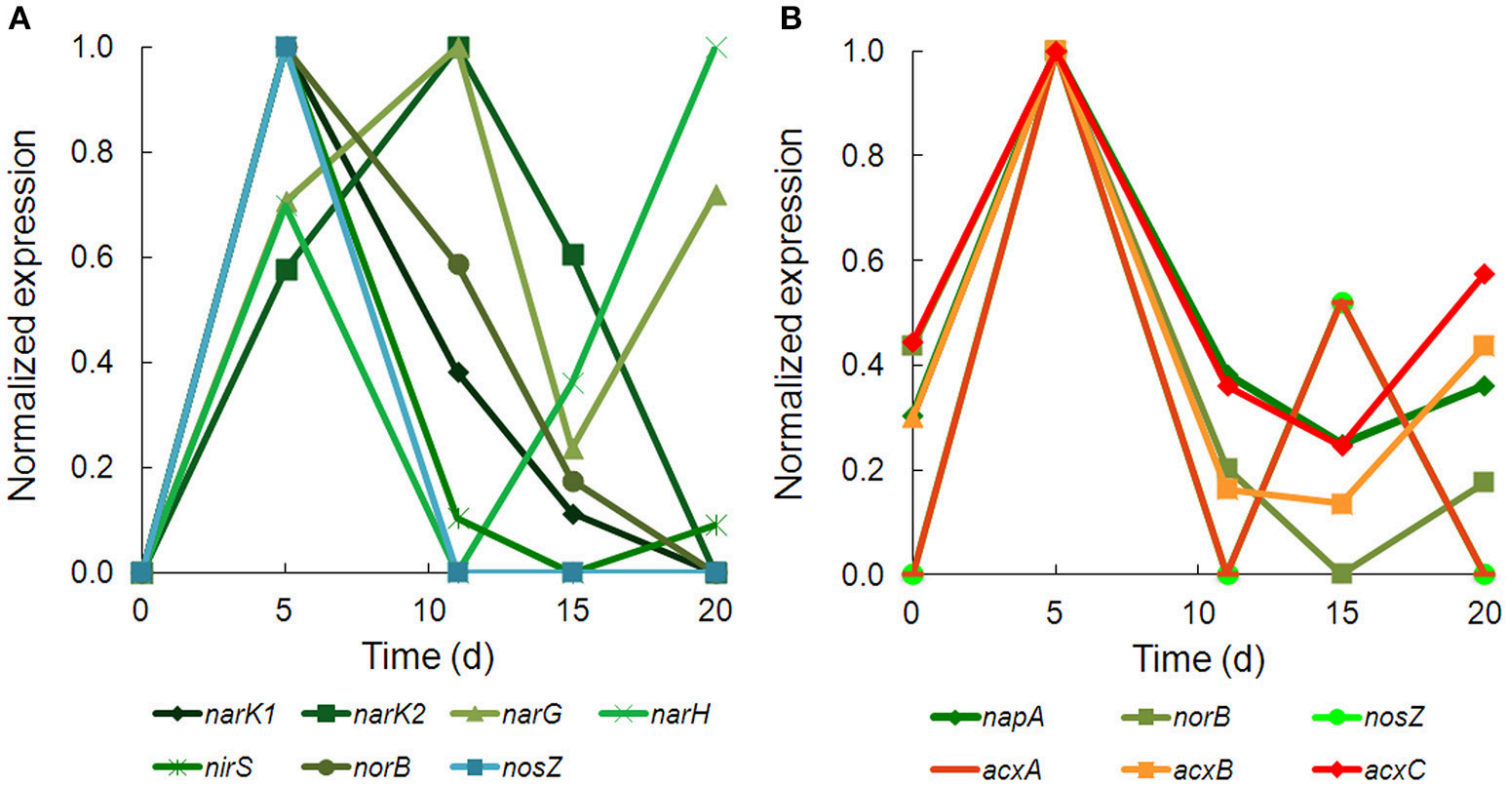
**Temporal expression of selected genes for (A)** denitrification in *Hydrogenophaga* b174 and **(B)** denitrification and acetone carboxylase in *Dechloromonas* b45. The expression (RPKM) has been normalized to the maximum for each gene.

There was strong transcriptional evidence of denitrification in *Hydrogenophaga* b174, with many of the structural genes and nitrate/nitrite transporters showing expression maxima at day 5 (Figure 5A; Supplementary Table 6). The genes were clustered on multiple scaffolds: membrane-bound respiratory nitrate reductase subunits and nitrate transporters formed a cluster (*narK*_1_*K*_2_*GH*) on one scaffold while cytochrome *cd*_1_ nitrite reductase (*nirS*) formed a cluster with other *nir* genes (*nirSMCFDLGHJN*) and nitric oxide reductase genes *norCBQ* and *norD* on a second scaffold. The scaffold containing the *nir* and *nor* genes had high synteny with *Hydrogenophaga* sp. LP0072, which shared 82, 87, and 86% translated sequence identity with *Hydrogenophaga* b174 *nirS, norB*, and *norC*, respectively (Supplementary Figure 2; Supplementary Table 7). This scaffold also included a 30S ribosomal protein gene with 95% translated sequence identity to that of *Hydrogenophaga* sp. T4. The gene encoding the enzyme that catalyzes the final step in denitrification, nitrous oxide reductase (*nosZ*), was present elsewhere in the genome. In addition to expressed denitrification, we found genes representing assimilatory nitrate reductase (*nasAB*) and a nitrite reductase (*norV*) that may be used to protect against oxidative stress (Silaghi-Dumitrescu et al., 2003). The assimilatory nitrate reductase transcripts had maximum expression at day 5, while *norV* expression was only detected on day 15. While it was unexpected to find active denitrification in a closed system with simulated groundwater containing no added nitrate, this finding was also supported by transcriptional evidence of denitrification in a *Dechloromonas* strain, b45, which expressed structural genes representing each step of denitrification: *napAB, nirS, norBC*, and *nosZ* (Figure 5B). Relatively low levels of nitrate were detected in the microcosms (~110 μM; Table 1), presumably released from the sediment; omics-based evidence of nitrification was not found, and would be very unlikely in this anoxic system. Interpretation of trends in the nitrate concentration data is confounded by relatively high standard deviations, particularly on days 15 and 20.

In addition to denitrification, we found genes encoding complete electron transport chain complexes I, II, III, IV, and V. Not all of these genes were expressed; the greatest expression was found for complex IV (cytochrome *cbb*_3_) genes, with five of the six subunits expressed. Relatively high expression of the low-oxygen-tension cytochrome oxidase characteristic of suboxic environments, cytochrome *cbb*_3_ (Pitcher and Watmough, 2004), was also found in conjunction with active denitrification in a previous metatranscriptomic study at the Rifle site (Jewell et al., 2016). Notably, the complex I genes *nuoABCDEFGHIJKLMN* shared a scaffold with the *nir* and *nor* denitrification genes described above.

#### Heterotrophic metabolism

Strong transcriptional evidence of a heterotrophic lifestyle was supported by expression of genes associated with use of numerous heterotrophic electron donors and carbon sources, including plant-derived biomass (glucose from cellulose, xylose from hemicellulose, and various phenolic compounds), fatty acids, and amino acids. Xylose degradation genes (*xylAB*) and xylose transporter genes (*xylFGH*) were organized in a single cluster and partially expressed; expression of glucose catabolism *via* the Entner-Doudoroff (ED) and glycolysis pathways was also observed. The presence of both ED and glycolysis pathways was surprising since, to date, there has been no documentation of glycolysis in the *Hydrogenophaga* genus. Indeed, the most recent entry for *Hydrogenophaga* in the Bergey's Manual (Willems and Gillis, 2009) only refers to a 1981 study (Lee and Schlegel, 1981) for heterotrophic metabolism in *H. pseudoflava* (formerly *Pseudomonas pseudoflava*). However, there are at least three *Hydrogenophaga* species (sp. Root209, sp. T4, and sp. LPB0072) with genes annotated as 6-phosphofructokinase, the enzyme unique to glycolysis (Bloxham and Lardy, 1973), in the GenBank non-redundant database. The translated sequences of the two genes unique to the ED pathway, phosphogluconate dehydratase and 2-keto-3-deoxy-6-phosphogluconate aldolase, were 86 and 93% identical to those of *Hydrogenophaga* sp. LPB0072, whereas the translated sequence of the gene unique to glycolysis, phosphofructokinase, was 91% identical to that in strain LPB0072. In addition to the two glucose catabolism pathways, genes for an incomplete anabolic pentose phosphate pathway were present. It is not unprecedented to have both catabolic pathways in the same organism, for example, a close relative, *Polaromonas* sp. JS666, has genes encoding both glycolysis and the ED pathway as well as an incomplete pentose phosphate pathway (Mattes et al., 2008).

Catabolism of plant-derived phenols was evidenced through expression of genes for ferulate degradation to vanillate (*faeA, ferAB, ligV*), pyrogallol (presumably gallate-derived) degradation to phloroglucinol, and further to oxaloacetate. Genes involved in fatty acid metabolism by β-oxidation were expressed along with numerous genes involved in metabolism of amino acids (arginine, proline, glutamate, aspartate, histidine, among others). Genes associated with metabolism of other N-containing compounds, namely urea, were highly expressed for urea hydrolysis (*ureABC*) and transport.

#### Chemolithoautotrophic metabolism

As the genus *Hydrogenophaga* was named for its ability to gain energy through hydrogen oxidation (Davis et al., 1969; Willems and Gillis, 2009), it is no surprise that transcripts associated with a membrane-bound uptake [NiFe] hydrogenase were identified in the metatranscriptome. *Hydrogenophaga* b174 had two [NiFe] hydrogenase clusters with the large and small subunits, *hupL* and *hupU*, respectively (note that the hydrogenase nomenclature is not standardized in the literature). The translated sequences for the pairs of small and large subunits were >95% identical to each other. These hydrogenases belong to Group 1 of the respiratory membrane-bound H_2_ uptake hydrogenases and the large subunit has the L2 signature subunit pattern (Vignais et al., 2001), except for the leading residue. Additional accessory and maturation proteins were found in clusters with these subunits, as well as elsewhere in the genome. While only one of the large subunits was expressed, and none of the small subunits, the accessory and maturation proteins *hupH* (k101_5515505_209934_6) and *hypF* (k101_2898432_117423_2) were among the most highly expressed ORFs in the metatranscriptome (Supplementary Table 8)

In addition to energy conservation from H_2_ oxidation, reduced sulfur compound oxidation and CO oxidation were also evident in the metatranscriptome. Transcripts from the S oxidation gene cluster, *soxCDc*_552_*YZAXB*, along with *soxR*, rhodanese, and *fcbbAB* genes, were detected. Transcripts were also detected from a gene cluster containing putative form I aerobic carbon monoxide dehydrogenase (CODH) subunits *coxS, coxM*, and *coxL*. This CODH is characterized for aerobic CO oxidation but has also been reported to generate reducing equivalents for nitrate reduction (King, 2006; Hoeft et al., 2007). The translated sequence of the large subunit, *coxL*, has the form II CODH active-site motif (AYRGAGR), distinguishing it from form I (King, 2003). However, this motif is found in a variety of enzymes with different substrate specificities and requires additional lines of evidence to confirm CO-specificity, including structural gene arrangement and the presence of accessory *cox* genes clustered with the structural genes (King and Weber, 2007). *Hydrogenophaga* b174 structural genes were clustered but not contiguous—*coxL* and *coxM* were separated by an ORF encoding a 37-amino acid peptide. This arrangement was not seen in any of the other *Hydrogenophaga* genomes examined. However, accessory proteins *coxFGEDM* were present in the cluster.

Chemolithoautotrophy fueled by H_2_, thiosulfate, and CO oxidation is widespread in the *Hydrogenophaga* genus (Graff and Stubner, 2003; Kämpfer et al., 2005; Yoon et al., 2008; Willems and Gillis, 2009). Oxidation of these compounds can also help fuel mixotrophic growth on organic compounds, giving the species a measurable growth advantage vs. purely heterotrophic growth (Kiessling and Meyer, 1982; Vanden Hoven and Santini, 2004). Beyond its role as an electron donor, CO can serve as an autotrophic carbon source through fixation of CO_2_,the product of its oxidation by CODH (Meyer and Schlegel, 1983).

### Metatranscriptome profiles during acetogenesis pulse

**Figure 6:**
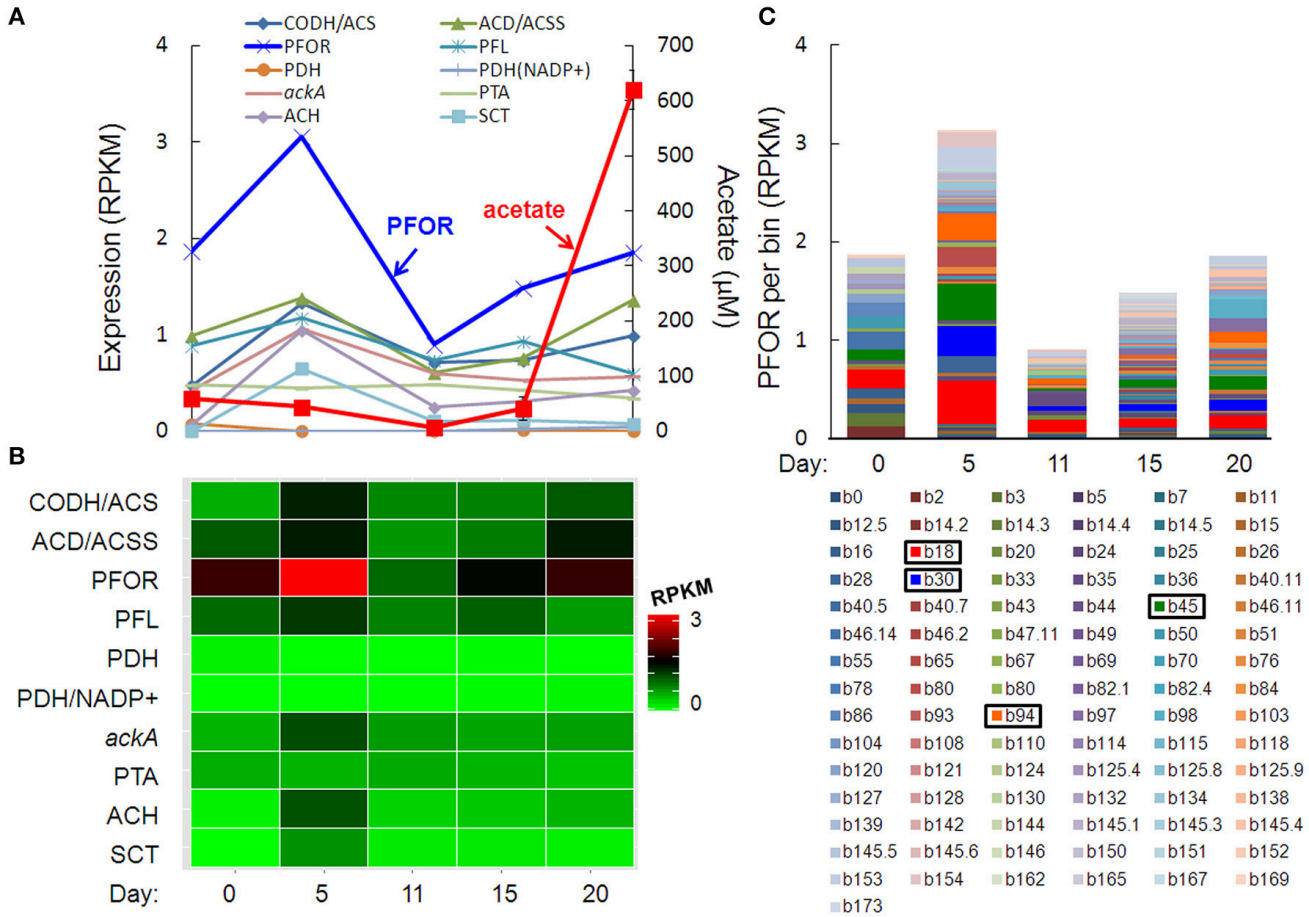
**Temporal expression profiles for transcripts potentially contributing to a pulse of acetogenesis after day 15: (A)** acetate-related gene expression and acetate concentration (red line), **(B)** heat map of the summed RPKM values from all contributing acetate-related transcripts, **(C)** PFOR RPKM values per bin, with more prominent bins highlighted in the key. Gene abbreviations: carbon monoxide dehydrogenase/acetyl-CoA synthase (CODH/ACS), acetyl-CoA decarbonylase/synthase (ACD/ACSS), pyruvate:ferredoxin oxidoreductase (PFOR), pyruvate formate-lyase (PFL), pyruvate dehydrogenase (PDH), NADP^+^-dependent pyruvate dehydrogenase (PDH/NADP+), acetate kinase (AckA), phosphate acetyltransferase (phosphotransacetylase, PTA), acetyl-CoA hydrolase (ACH), and acetate:succinate CoA-transferase (SCT).

The pulse of acetogenesis beginning at day 15 was apparently not linked to the metabolism of just one or two dominant taxa, but rather, a relatively large number of taxa with acetate-generating metabolisms, particularly the autotrophic, carbon-assimilating Wood-Ljungdahl pathway and multiple fermentation pathways (Figure 6). Highly expressed genes in acetate- (and acetyl-CoA-) producing pathways included carbon monoxide dehydrogenase/acetyl-CoA synthase (different than the “aerobic” CODH mentioned earlier), acetyl-CoA decarbonylase/synthase, pyruvate:ferredoxin oxidoreductase (PFOR), pyruvate formate-lyase, pyruvate dehydrogenase, NADP^+^-dependent pyruvate dehydrogenase, acetate kinase, phosphate acetyltransferase (a.k.a. phosphotransacetylase), acetyl-CoA hydrolase, and acetate:succinate CoA-transferase (Figures 6A,B). Of these, PFOR was the most prominent in the metatranscriptome, with PFOR expression relatively evenly distributed over a large number of taxa, but with more significant contributions from a small number of taxa, notably, *Dechloromonas* b45 and the multi-taxa bins b18, b30, and b94 (Figure 6C, Supplementary Table 9).

### Characterization of most highly expressed genes

**Figure 7:**
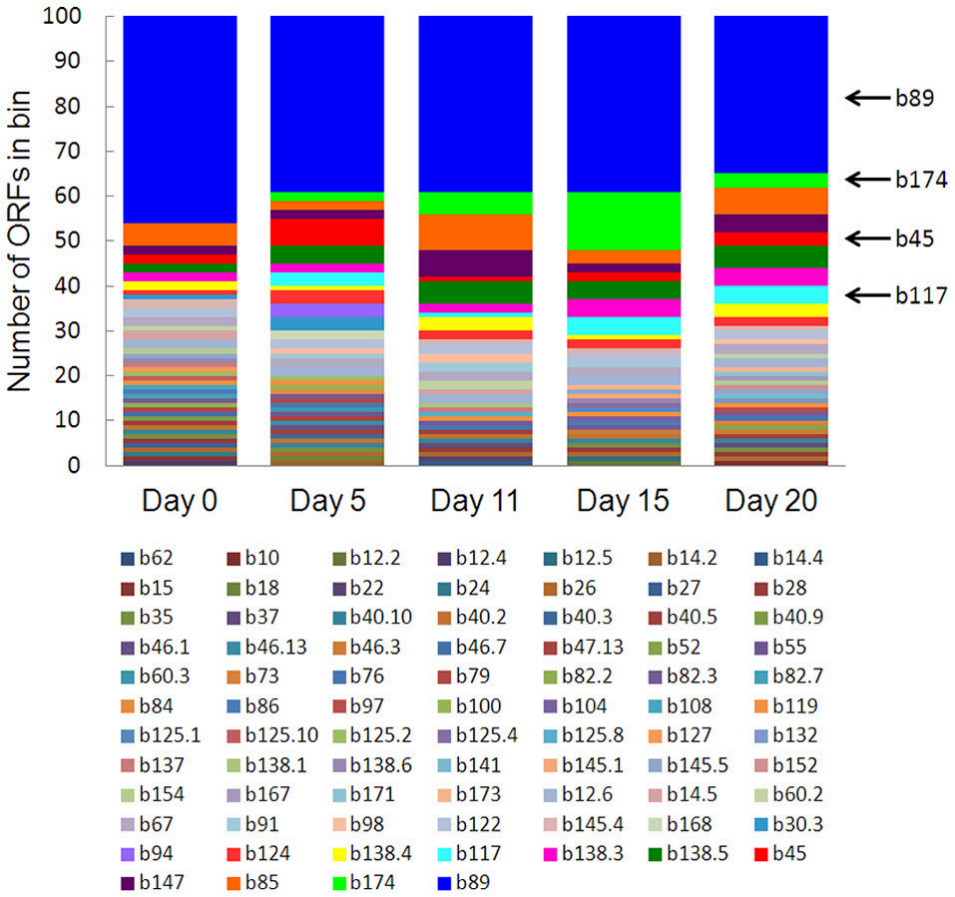
**The number of ORFs from contributing bins represented in the top 100 transcripts at each time point**. Bin 89 represents *Ca*. Bathyarchaeota.

Eighty-one bins contributed to the 100 most highly expressed (≥99th percentile) genes over the 20-day study; three taxonomic groups, *Dechloromonas* (b45), *Ca*. Bathyarchaeota (b89), and *Hydrogenophaga* (b117, b174) collectively accounted for approximately half (45–56%) of the top 100 transcripts (Figure 7). A full list of the top 100 transcripts per day can be found in Supplementary Table 8. Three types of genes were consistently included among the top 100 transcripts: hydrolases (most notably cell wall-associated hydrolases), acetone carboxylase subunits, and transposases.

**Figure 8:**
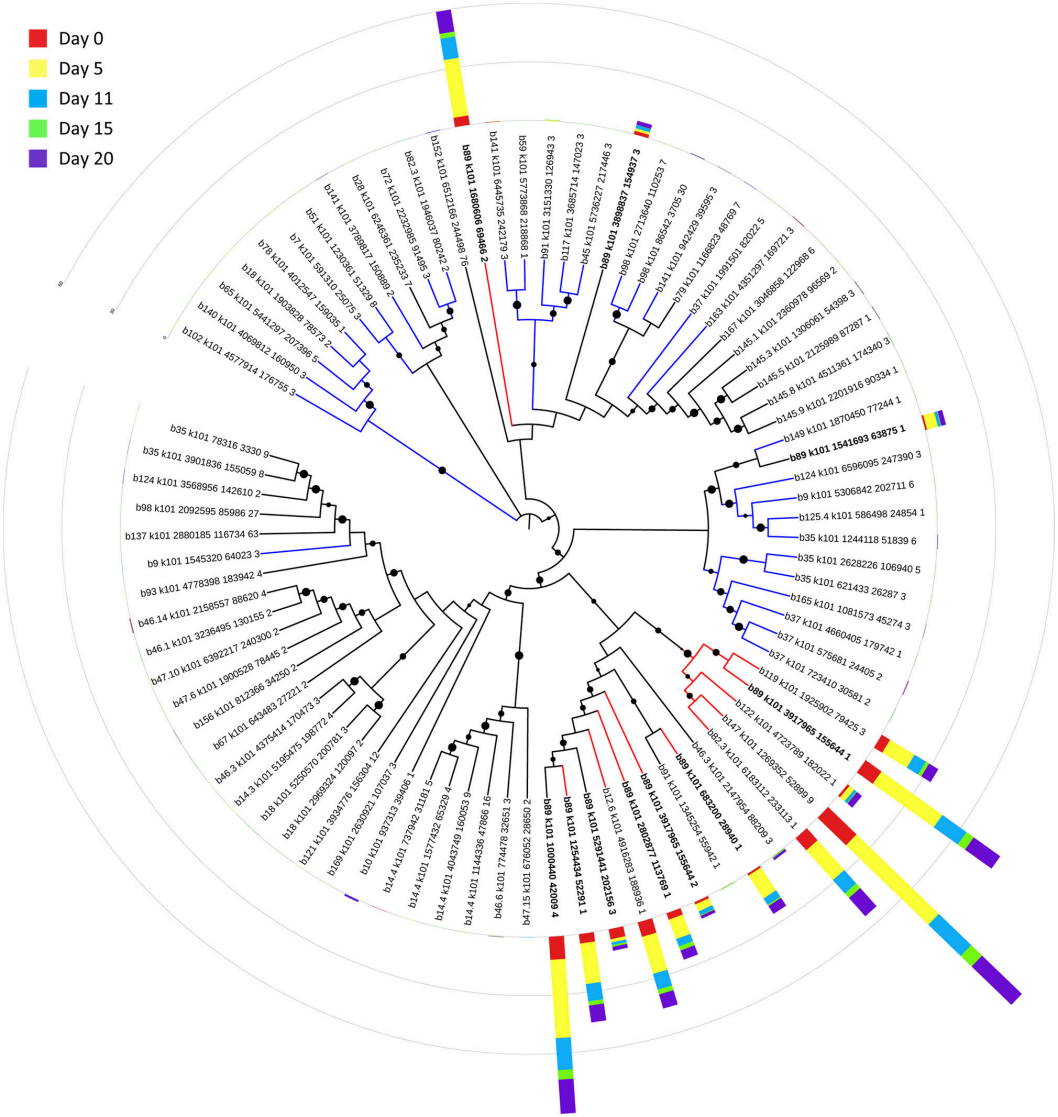
**Phylogenetic tree of expressed hydrolases**. Red lines represent ORFs from the top 100 transcripts annotated as cell wall-associated hydrolases. Blue lines represent cell wall-associated hydrolases with a peptidoglycan-targeting catalytic domain. ORFs from *Ca*. Bathyarchaeota b89 are shown in boldface. Local support values (50–100%) for nodes are represented as circles scaled from small to large. The expression (RPKM) for each ORF at each time point is represented by stacked histograms encircling the tree. The RPKM values, annotations, and sequence identities for each ORF in this tree can be found in Supplementary Table 9. The hydrolase sequences were aligned with Clustal Omega (Sievers et al., 2011) and trimmed and visualized with Jalview (Waterhouse et al., 2009).

Bin 89, classified as *Ca*. Bathyarchaeota, was the best represented bin amongst the top-100 transcripts at all sampling times, ranging from 35 to 49% of the top 100 transcripts (Figure 7). *Ca*. Bathyarchaeota is a recent reclassification of the archaeal Miscellaneous Crenarchaeota Group (MCG) (Meng et al., 2014) and some members are thought to live a scavenging lifestyle, consuming plant-derived carbohydrates and detrital proteins (Lazar et al., 2016). This was reflected by *Ca*. Bathyarchaeota b89, which expressed numerous hydrolases, including 10 in the top 100 most highly expressed ORFs (Supplementary Table 8). Four of these hydrolases were annotated as cell wall-associated hydrolases; the translated sequence with the best identity (99%), but low coverage (35%), belonged to *Sulfurospirillum multivorans* DSM 12446, whereas the one with the best identity and coverage (72 and 73%, respectively) belonged to *Peptoclostridium difficile* CD7. Cell wall-associated hydrolases target peptidoglycan and can have multiple functions, often for the same enzyme (Wyckoff et al., 2012), such as cell growth (e.g., Höltje, 1995), physical predation on bacterial cells for nutrients (e.g., Lerner et al., 2012), and community restructuring by selective targeting of individual species (Hood et al., 2010; Schwarz et al., 2010). The *Ca*. Bathyarchaeota hydrolases did not, however, possess the conserved NlpC/P60 catalytic domain associated with hydrolases that target peptidoglycan (Margot et al., 1999; Xu et al., 2009). Cell wall-associated hydrolases belonging to other taxa in the metatranscriptome did contain this NlpC/P60 domain. A phylogenetic tree of all translated hydrolase transcripts showed that the majority of the top 100 hydrolases clustered together and apart from the hydrolases containing the peptidoglycan-targeting domain (Figure 8, Supplementary Table 9). While the substrate(s) that these *Ca*. Bathyarchaeota hydrolases are targeting is unknown, the very high relative expression of the transcripts suggests that they play a significant role in carbon transformation in this system.

Acetone carboxylase subunits (*acxA, acxB, acxC*) with 97–99% translated sequence identity to those of *Dechloromonas* spp. were among the most highly expressed ORFs in the metatranscriptome (Supplementary Table 8). Acetone carboxylase can be used by bacteria to assimilate CO_2_ under anaerobic conditions and channel acetone into central carbon metabolism (*via* acetoacetate). It appears that *Dechloromonas* may have coupled denitrification to acetone carboxylation, particularly at day 5, when expression of multiple denitrification and acetone carboxylase genes were all at their maxima (Figure 5B). Such nitrate-dependent metabolism of acetone has been in observed in other denitrifiers (Platen et al., 1990; Dullius et al., 2011; Oosterkamp et al., 2015), but not in *Dechloromonas*. Acetone carboxylase belonging to Chloroflexi was observed at this field site in a previous metagenomics study and was noted as potential source of acetate (*via* acetyl-CoA) in the environment (Hug et al., 2013). To explain the presence of acetone in this system, we looked for transcriptional evidence of acetoacetate decarboxylase, and found these transcripts in two Chloroflexi bins (k101_4019724_159251_3 in b46.3 and k101_1257655_52416_1 in b46.13) and in a Bacteroidetes/Chlorobi bin (k101_5859895_221904_4, b124), albeit with low translated sequence identities to a well-characterized acetoacetate decarboxylase sequence from *Clostridium acetobutylicum* (23–27%) (Gerischer and Dürre, 1990).

Transposases belonging to a variety of taxonomic bins were prominent in the top 100 expressed transcripts. This is unsurprising, since a survey of protein-coding genes in data repositories (e.g., SEED, RAST) identified transposases as the most abundant genes in nature (Aziz et al., 2010), a claim that has been supported by metagenomic and metaproteomic studies in diverse environments (Ram et al., 2005; Brazelton and Baross, 2009; Kleiner et al., 2013).

## Discussion

NRZs have been characterized as biogeochemical hot spots at the Rifle site (and elsewhere) and have been associated with a relative enrichment of organic matter and anaerobic microbial metabolism, including both fermentative and respiratory [e.g., sulfate and Fe(III) reduction] pathways, based on geochemical and metagenomic data (Campbell et al., 2012; Hug et al., 2013; Janot et al., 2015). Here, we further investigated dynamic metabolic activity in NRZ hot spots at the Rifle site by integrating strain-specific metatranscriptomic and metagenomic data with geochemical data in anaerobic microcosms in which NRZ aquifer sediment served as the sole source of microorganisms and electron donors.

While metatranscriptome data substantiated major trends indicated by geochemical data (namely, DOM mineralization and acetogenesis), they also provided some results that were unexpected in light of previous characterizations of NRZs, including the following: (1) in addition to the expected fermentative metabolism, which was observed, two taxa (*Hydrogenophaga* b174 and *Dechloromonas* b45) showed strong evidence of denitrification, (2) in addition to heterotrophic metabolism, which was expected and observed, both chemolithoautotrophic metabolism (with H_2_, CO, and S species as electron donors) and heterotrophic metabolism were active in *Hydrogenophaga* b174, a strain that accounted for the largest portion of the metatranscriptome observed in the study, (3) in addition to the expected use of plant-derived biomass as a carbon source and electron donor, which was observed, evidence of cell wall-associated hydrolases (e.g., targeting peptidoglycan) and acetone carboxylase among the most highly expressed genes in the study suggest other important energy and carbon sources, including acetone and potentially bacterial biomass, and (4) *Ca*. Bathyarchaeota, particularly bin 89, had a disproportionately large presence in the metatranscriptomes of all samples, although this phylum is best known for its occurrence in marine, estuarine, and freshwater sediments (Meng et al., 2014; Evans et al., 2015; He et al., 2016; Lazar et al., 2016). Below, we elaborate on these findings in the context of how they build and expand on knowledge from existing studies of Rifle NRZs.

Nitrate reduction has been postulated to play a role in NRZ metabolism (Janot et al., 2015) but this activity had not been documented in Rifle NRZs before. The metabolic potential for reduction of NOx species has been suggested by metagenomic analysis of NRZ sediments (e.g., from Rifle well D04, located ca. 4.4 m from well CMT-03). For example, nitrite reductases (*nirK* and *nrfA*) were reported in some reconstructed Chloroflexi genomes in well D04 sediments (Hug et al., 2013) and an apparent NXR complex (first described as a nitrite:nitrate oxidoreductase in anammox bacteria) was reported in the reconstructed RBG-1 genome in these sediments (Castelle et al., 2013). We found transcriptional evidence of active denitrification in *Hydrogenophaga* b174 and *Dechloromonas* b45 based on expression of canonical denitrification genes (e.g., Figure 5). In the experimental context of the present study (e.g., with a complex community and undefined mass balance for major elements), it is difficult to definitively identify the electron donors that were coupled with denitrification in *Hydrogenophaga* b174. However, for some observed chemolithoautotrophic reactions, such as S-compound oxidation and “aerobic” carbon monoxide dehydrogenase, coupling to nitrate reduction seems probable. This is because S-compound oxidation and “aerobic” carbon monoxide dehydrogenase would be energetically constrained if coupled with electron acceptors less favorable than O_2_ or nitrate [e.g., Fe(III) or sulfate]; indeed, they have only been documented to be coupled with O_2_ or nitrate in chemolithoautotrophic, mesophilic prokaryotes (this excludes anaerobic photolithotrophs, which are not relevant in our light-protected experimental system) (Dobbek et al., 1999; Friedrich et al., 2005; King, 2006; Hoeft et al., 2007; Ghosh and Dam, 2009). Since geochemical evidence strongly indicates that CMT-03 microcosms were strictly anaerobic, it is reasonable to deduce that some, perhaps all, of the active chemolithoautotrophic metabolism of *Hydrogenophaga* b174 was coupled to nitrate reduction/denitrification.

Rifle aquifer studies from non-NRZ regions also have reported the emergence of *Hydrogenophaga* spp., although this was typically in the context of chemolithotrophy under aerobic/microaerophilic (not nitrate-reducing) conditions. For example, in flow-through column studies with reduced Rifle aquifer sediment incubated under microaerophilic conditions, *Hydrogenophaga* spp. constituted up to 98% of the groundwater community and Fe, S, and U oxidation were observed (N'Guessan et al., 2010); other β-proteobacterial chemolithoautotrophs, such as *Thiobacillus* spp., were also prominent in the system and may have accounted for some of this activity. In another study, *Hydrogenophaga* sp. accounted for 8% of the *in situ* microbial community in biofilms associated with aquifer sediments after acetate amendment (Williams et al., 2013). Finally, a Fe(II)-oxidizing *Hydrogenophaga* strain (P101) was isolated from Rifle groundwater under microaerophilic conditions (Chan, 2015); this strain had a facultative mixotrophic phenotype, in that it could grow on complex, low-concentration organic medium as well as ferrous minerals like FeS and FeCO_3_.

To some extent, *Hydrogenophaga* b174 and other microbes described in this study expressed components of the heterotrophic metabolic potential that have been described in previous metagenome studies of Rifle NRZ sediments, such as fermentative metabolism of plant-derived organic matter and acetogenesis (e.g., Hug et al., 2013). For example, we have documented expression of fermentation of plant-derived glucose *via* glycolysis and PFOR (e.g., Figures 4, 7), metabolism of plant-derived phenolic compounds (e.g., ferulate and gallate), homoacetogenesis *via* the Wood-Ljungdahl pathway (carbon monoxide dehydrogenase/acetyl-CoA synthase), and beta-oxidation of fatty acids, all of which have been encoded in reconstructed genomes of selected Rifle NRZ microbes (e.g., Hug et al., 2013). However, the very high expression levels (≥99th percentile) of acetone carboxylase and cell wall-associated hydrolases were unexpected and have implications for heterotrophic metabolism in this NRZ system. In *Dechloromonas* b45, expression of acetone carboxylase and denitrification genes were highly correlated (Figure 5), and it is possible that acetone carboxylase was being used to assimilate CO_2_ and channel acetone into central carbon metabolism (*via* acetoacetate and eventually acetyl-CoA) under denitrifying conditions (Platen and Schink, 1990). A potential source of acetone in this microbial community (*via* acetoacetate decarboxylase) was discussed earlier. Alternatively, it is also possible that the acetone carboxylase reaction could be run in reverse (as demonstrated *in vitro*; Platen and Schink, 1990), which would yield energy in the form of ATP from acetoacetate and ADP (Equation 1 for the forward reaction; Platen and Schink, 1990):

(1) $$\begin{array}{l} {\text{CH}}_{3}\text{​​}-\text{​CO​}-\text{​}{\text{CH}}_{3}\;(\text{acetone})\;+\;{\text{CO}}_{2}(\text{aq})\;+\text{ ATP}\\ \;+\;{\text{H}}_{2}\text{O }\to \;{\text{CH}}_{3}\text{​}-\text{​CO}-\text{​}{\text{CH}}_{2}\text{​}-\text{​}{\text{COO}}^{-}(\text{acetoacetate})\\ \;+\;{\text{H}}^{+}\;+\text{ ADP }+\;{\text{P}}_{\text{i}} \end{array}$$

The reverse reaction is endergonic at pH 7 and standard-state conditions (Platen and Schink, 1990), but could become exergonic at plausible (non-standard-state) substrate and product concentrations. Theoretically, sources of acetoacetate for the reverse reaction could include acetoacetyl-CoA (e.g., from beta-oxidation) and conversion of acetoacetyl-CoA to acetoacetate *via* a CoA-transferase reaction. To our knowledge, this pathway involving acetone carboxylase (in reverse) has not been documented *in vivo*, but it provides a possible explanation for the very high expression of acetone carboxylase in a system that may not have a copious source of acetone. These potential heterotrophic reactions, along with potential scavenging of bacterial biomass and other implications of highly expressed cell wall-associated hydrolases (discussed earlier) provide further insights into what is known about microbial metabolism in NRZs.

Further research will build on our emerging understanding of biogeochemical dynamics in NRZs. Specifically, an ongoing field study involves the release of nitrate into a subsurface NRZ at the Rifle site, which will provide more information on N cycling in these zones, where nitrate reduction and denitrification appear to be important, and will also provide an interesting comparison for a previous nitrate release at an area that did not include NRZs (Jewell et al., 2016).

## Author contributions

HB and TJ conceived of and designed the experiments. TJ (primarily), HB, KW (field component), MB (TOC and isotopic data), and RC (respirometer data) performed the experiments. TJ and HB analyzed the data. UK performed co-assembly, binning, annotation, and mapping of transcriptional data. The manuscript was written by TJ and HB (primarily) and all authors contributed to refining the text.

## Funding

This work was supported as part of the Subsurface Biogeochemical Research Scientific Focus Area funded by the U.S. Department of Energy, Office of Science, Office of Biological and Environmental Research under Award Number DE-AC02-05CH11231.

### Conflict of interest statement

The authors declare that the research was conducted in the absence of any commercial or financial relationships that could be construed as a potential conflict of interest.
