# Supplementary figures and images for: Metatranscriptomic Analysis Reveals Unexpectedly Diverse Microbial Metabolism in a Biogeochemical Hot Spot in an Alluvial Aquifer
Source: Front Microbiol. 2017 Jan 25;8:40. doi: 10.3389/fmicb.2017.00040 (PMC5264521; doi:10.3389/fmicb.2017.00040)

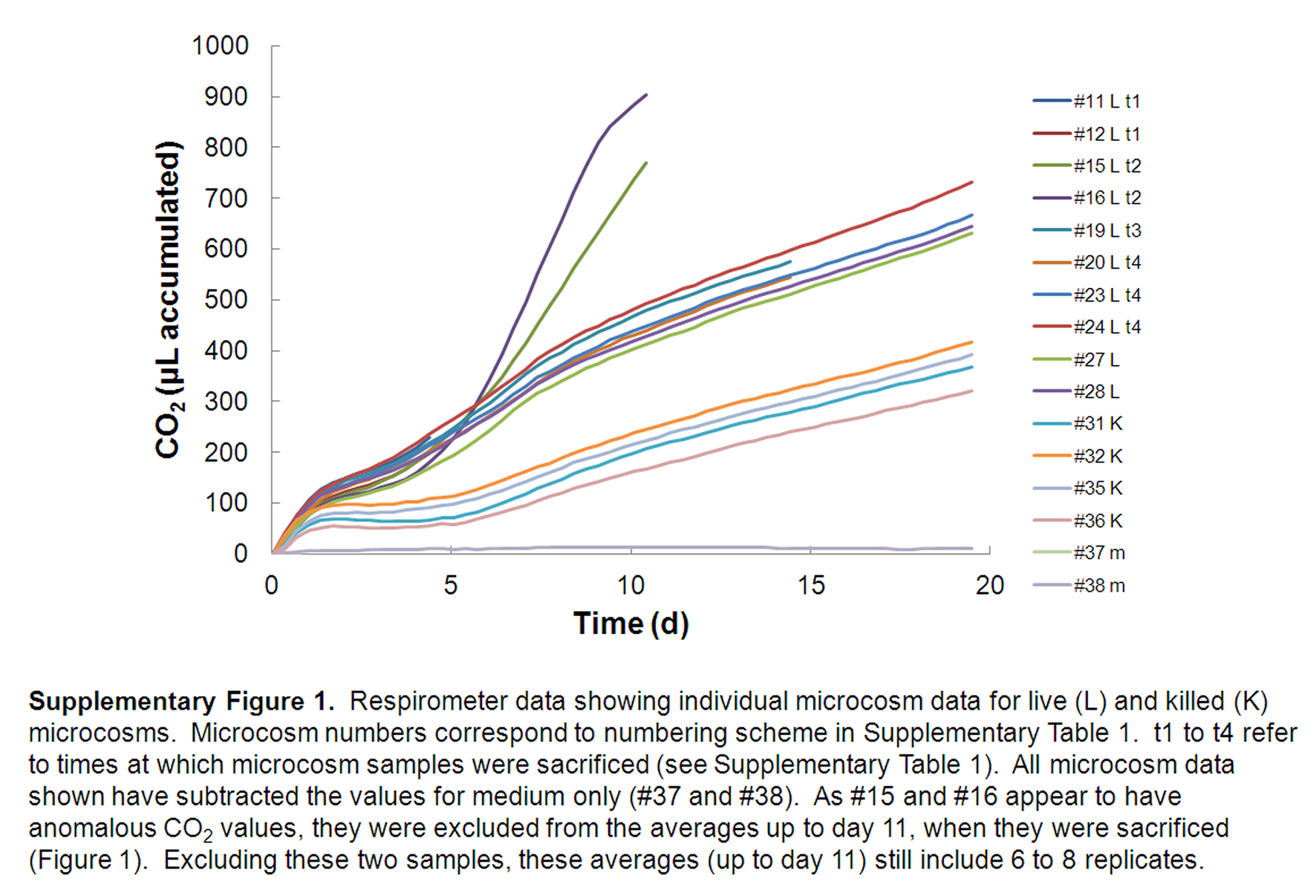

Supplement: Supplementary file 10 [file Image1.TIF]

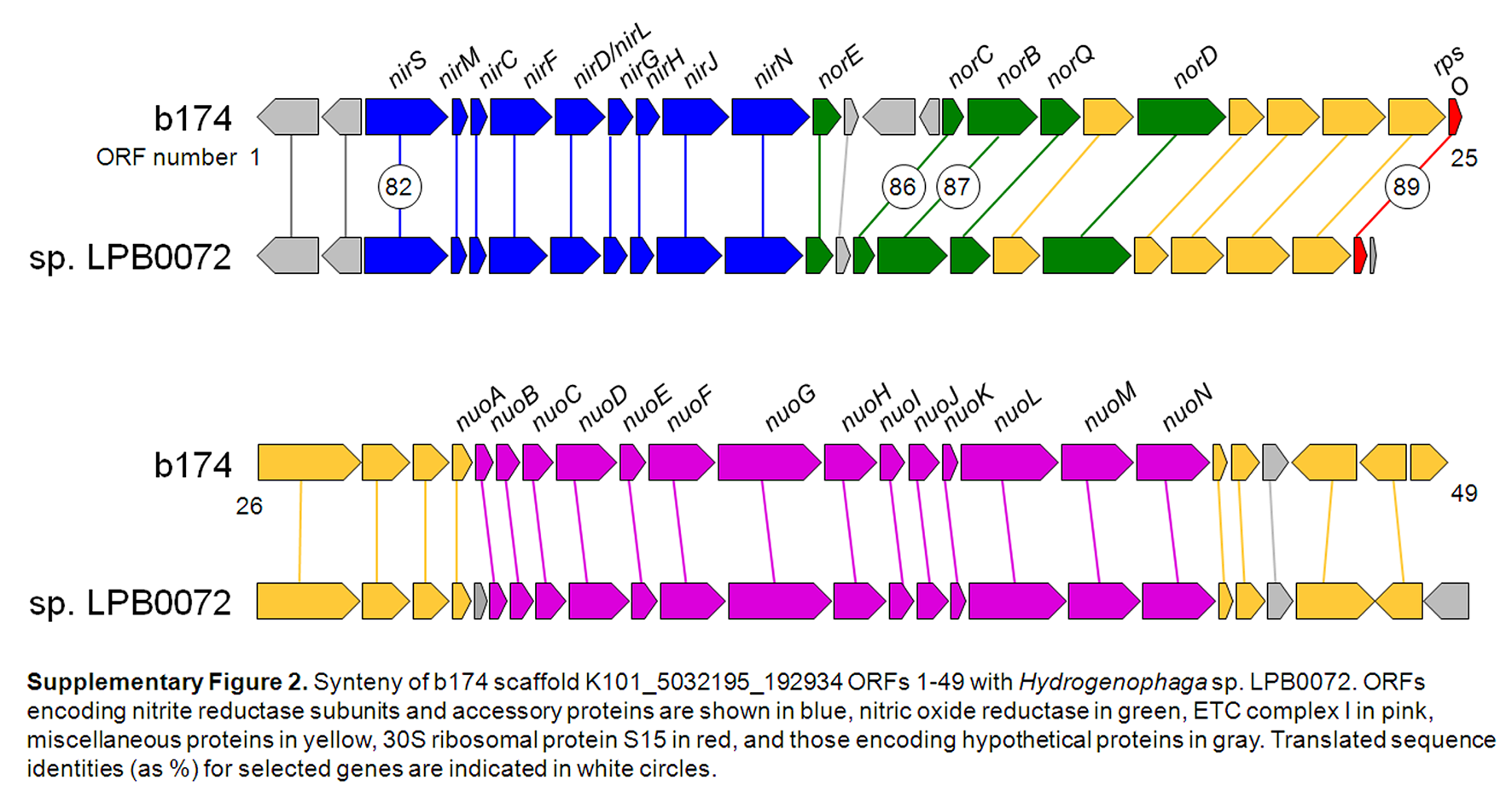

Supplement: Supplementary file 11 [file Image2.TIF]
